# Topical tacrolimus for high-risk corneal transplantation: a randomized, clinical trial

**DOI:** 10.1186/s12886-024-03506-6

**Published:** 2024-06-12

**Authors:** Jun Shimazaki, Daisuke Tomida, Yukari Yagi-Yaguchi, Yoshiyuki Satake, Takefumi Yamaguchi

**Affiliations:** Department of Ophthalmology, Tokyo Dental College Ichikawa General Hospital, 5-11-13 Sugano, Ichikawa, 272-8513 Chiba Japan

**Keywords:** Penetrating keratoplasty, Tacrolimus, High-risk patients, Randomized clinical trial

## Abstract

**Background:**

The prevalence of rejection is 10–30% in penetrating keratoplasty (PKP) case, and the rate is higher in cases of high-risk patients. Although using topical corticosteroids is a standard method for management the rejection of post-PKP patients, it may not be sufficiently potent in high-risk patients. Topical administration of tacrolimus (TAC) may be effective in suppression rejection after corneal transplantation. This study aimed to investigate the efficacy and safety of topical TAC in high-risk PKP patients in Japan.

**Methods:**

This study was a single centre, single-blinded, randomized controlled trial. Patients with a history of PKP, graft rejection, atopic dermatitis, or deep corneal neovascularisation who underwent PKP were enrolled. They were randomly assigned to receive 0.1% TAC ophthalmic suspension or artificial tear (AT) up to week 52 after surgery. All participants received 0.1% betamethasone up to week 13 after surgery then they received 0.1% fluorometholone up to week 52. The incidence of immunological rejection during the observation period was the main outcome measure in this study.

**Results:**

Thirty patients were enrolled in this study, and 12 eyes in the TAC group and 13 eyes in the AT group completed the study, respectively. Five out of 30 patients discontinued participation after providing informed consent. No serious adverse effects were developed in patients who received 0.1% TAC ophthalmic suspension. No rejection episodes occurred in the TAC group, while one eye in the AT group had rejection. Graft clarity, best spectacle-corrected visual acuity, intraocular pressure, and corneal endothelial cell density were not significantly different between the TAC and AT groups.

**Conclusion:**

Our results demonstrated that good tolerability of 0.1% TAC ophthalmic suspension. However, we failed to demonstrate its efficacy in preventing immunological rejection in high-risk patients undergoing PKP.

**Trial registration:**

This study was first registered in the University Hospital Medical Information Network (UMIN000029669, Date of registration: November 1, 2017). With the enforcement of the Clinical Trial Act in Japan, the study re-registered in the Japan Registry of Clinical Trials (jRCTs031180342, Date of registration: March 18, 2019).

## Background

Management of postoperative immunological rejection is a key factor for successful penetrating keratoplasty (PKP). While the prevalence of rejection is 10−30% in uncomplicated PKP cases, the rate is considerably higher in cases of high-risk PKP [1–4]. Various types of immunosuppressants have been introduced to control post-PKP rejection. Topical corticosteroids remain the gold standard; however, they may not be sufficiently potent in high-risk cases. In addition, prolonged use of corticosteroid eye drops can result in vision-threatening complications, such as cataract, glaucoma, and infectious keratitis. The efficacy of systemic administration of cyclosporine A (CsA) has been well studied, but the results have been inconsistent [5–13]. Mycophenolate mofetil has also been used, with variable results [6, 13–16]. Systemic tacrolimus (TAC) may be an alternative treatment approach, and some encouraging results have been reported. Our group demonstrated that systemic TAC was superior to CsA, with higher efficacy in preventing irreversible rejection and fewer side effects [17].

Topical administration of immunosuppressives is theoretically feasible and preferred, particularly because the topical administration seldom elicits systemic side effects. However, the lack of commercially available topical immunosuppressives for PKP, other than corticosteroids, is a major clinical problem. While several reports mentioned about efficacy of topical TAC for prevention of immunological rejection in other country [18–20], there is no study demonstrated efficacy and safety of topical TAC for high-risk PKP patients in Japan. In the present study, we aimed to investigate the efficacy and safety of commercially available topical TAC in high-risk patients undergoing PKP. From an ethical perspective, the basic treatment with corticosteroids was needed for high-risk PKP patients. Thus, we set TAC as an add-on drug, and for adjustment number of drugs, artificial tear was chosen as control drug compared to TAC.

## Methods

### Study design

This was a randomised, single-blind, 52-week, two-arm, single-centre clinical trial conducted from 24 November 2017 (first patient enrolment) to 5 February 2021 (last patient’s last visit) at the Tokyo Dental College Ichikawa General Hospital, a tertiary referral hospital in the Tokyo metropolitan area.

The inclusion criteria were age ≥ 20 years and met criteria for high-risk keratoplasty: history of PKP, history of graft rejection, deep corneal vascular invasion (more than two quadrants), or keratoconus with atopic diseases. The exclusion criteria were as follows: contraindications for TAC ophthalmic suspension, betamethasone phosphate sodium ophthalmic solution, or fluorometholone ophthalmic suspension; diabetes with poor glycaemic control; glaucoma with poor intraocular pressure (IOP) control; ocular infections; indicated limbal transplantation; malignancy; serious liver, kidney, cardiovascular, or endocrine disorders; pregnancy; use of topical/systemic steroids and/or immunosuppressants within 4 weeks before the study; and planned use of steroids or immunosuppressants other than the study drugs.

Immunological rejection may develop in up to 73% of cases following high-risk PKP [21]. It was reported that TAC eye drops suppressed irreversible rejection by approximately 56% compared to conventional treatment [18]. Assuming that the addition of TAC ophthalmic suspension will reduce the incidence of rejection from 73 to 32%, the number of patients required to verify the rejection suppression effect at a significance level of 5.0% on the two sides and a power of 80% was calculated as 22 per group. Among the patients undergoing PKP at our department, approximately 20 per year classify as high-risk patients. Therefore, in the present study, we set the sample size at 15 subjects per group who could be enrolled for approximately 1.5 years, which is close to the estimated number of cases.

### Treatment schedule

Treatment was started on the day after surgery. Patients visited the facility every thirteen week until week 52 for examination of this study. They were randomly assigned in a 1:1 ratio to receive TAC ophthalmic suspension BID (TAC group; 0.1% TALYMUS® ophthalmic suspension, Senju Pharmaceutical Co., Ltd., Osaka, Japan) or artificial tears BID (AT group; ARTIFICIAL TEAR MYTEAR® ophthalmic solution, Senju Pharmaceutical Co., Ltd., Osaka, Japan) from soon after PKP surgery to week 52. All patients received betamethasone ophthalmic solution QID (0.1% betamethasone phosphate Na·PF® solution, Rohto Nitten Co., Ltd. Nagoya, Japan) in the first 13 weeks, and fluorometholone ophthalmic suspension QID (Flumetholon® 0.1%, Santen Pharmaceutical Co., Ltd., Osaka, Japan) from week 13 to week 52.

To ensure researcher blindness, all study drugs were placed in an opaque blank paper box and sealed. The drug allocation manager prepared an allocation table in which 0.1% TALYMUS® ophthalmic suspension or ARTIFICIAL TEAR MYTEAR® ophthalmic solution was assigned to each sequence. The allocation table was sealed and stored by the drug allocation manager until the end of the observation period.

### Study endpoints

The primary endpoint was the difference in the incidence of rejection between the two treatment groups during the observation period. Endothelial rejection was defined as the development of acute onset of corneal oedema in the operated eye associated with inflammatory response in the anterior chamber and/or development of keratoprecipitates at the oedematous region.

Secondary endpoints included the graft survival rate, best spectacle-corrective visual acuity (BSCVA), and corneal endothelial cell density (ECD). The BSCVA was determined using the Snellen chart, and decimal values were converted to the logarithm of the minimal angle of resolution units for statistical analysis. For analysis, finger counting, hand movements, and light perception were converted to 0.004, 0.002, and 0.001, respectively. Corneal ECD was measured using a non-contact specular microscope (EM-4000; Tomey, Aichi, Japan). The percentage decrease in ECD was calculated using the following equation: (preoperative donor corneal ECD- postoperative ECD)/preoperative ECD × 100.

Safety endpoints included the following: IOP changes, corneal epithelial damage, and ocular adverse events. IOP was measured three times using a Tono-Pen® XL Applanation Tonometer (Reichert Technologies, Depew, New York, USA), and the mean measured value was calculated. Cross-tabulation was conducted on the number of cases with or without IOP increase of ≥ 5 mmHg at week 52 after surgery. Corneal epithelial damage was assessed semi-quantitatively as follows. The density of superficial epitheliopathy was assessed using the fluorescein staining test, with results ranging from grade 0 (no staining) to a maximum of 3 (severe staining). The area of punctate staining was also assessed and ranged from 0 (no staining) to 3 (stained area covering the entire cornea). All adverse reactions were assessed and coded using terminology from the Medical Dictionary for Regulatory Activities version 24.1.

### Surgical methods

All surgeries were performed under retrobulbar anesthesia with lidocaine 2.0% plus epinephrine. Donor corneas were obtained from the Cornea Center Eye Bank in Tokyo Dental College or an eye bank in the United States (CorneaGen, Seattle, WA, USA), and all met the donor quality criteria of the Eye Bank Association of America. Human leukocyte antigen matching was not performed. In most cases, donor corneas were 7.5 mm in diameter. During surgery, 0.25-mm oversized donor corneas were punched out using a Barron donor punch (Katena Products, Inc., Denville, New Jersey, USA) and secured to the recipient’s eye after excision of the abnormal host cornea using the Hessbarg–Barron trephine (Katena Products, Inc.). Single, continuous, or interrupted 10 − 0 nylon sutures or 16-bite interrupted sutures were used. Subconjunctival betamethasone (2 mg) was administered at surgery completion.

### Statistical analyses

The full analysis set included all randomised patients who received the study drug at least once, and the safety set included all patients with one or more data. For the analysis, missing or ineligible data were excluded. At week 26, all data of two patients in the TAC group were excluded because of inadequate switching of steroids or missed visits due to the threat of coronavirus infection. At week 39, all data of three patients in the AT group were excluded because of concomitant use of prohibited drugs, missed visits due to the threat of coronavirus infection, or dropout. At week 52, all data of one patient in the TAC group and four patients in the AT group were excluded because of concomitant use of prohibited drugs, interruption of administration, or dropout. In the TAC group, corneal ECD data were missing in two cases at week 39 and in one case at weeks 13 and 52 each because they were impossible to measure.

Data are presented as the mean ± standard deviation or median. Graft survival rates were calculated using Kaplan-Meier analysis. The log-rank test was employed to compare the incidence of rejection and graft survival rates between the groups. The log-rank test to determine difference between groups was one-tailed, and was conducted significance of alpha level was set at < 0.05. Mann-Whitney two sample u test was performed to compare continuous variables of BSCVA and corneal ECD between baseline and each observation time point (weeks 13, 26, 39, and 52). Fisher’s exact test was used for comparison of categorical data of IOP, corneal epithelial damage, and adverse events. These statistical analyses to determine difference between groups were two-tailed, and were conducted significance of alpha level was set at < 0.05. All analyses were performed using SAS version 9.4 (SAS Institute Inc., Cary, NC, USA), and all graphs were made using JMP version 15.2.1 (SAS Institute Inc., Cary, NC, USA).

## Results

### Patient characteristics

**Fig. 1:**
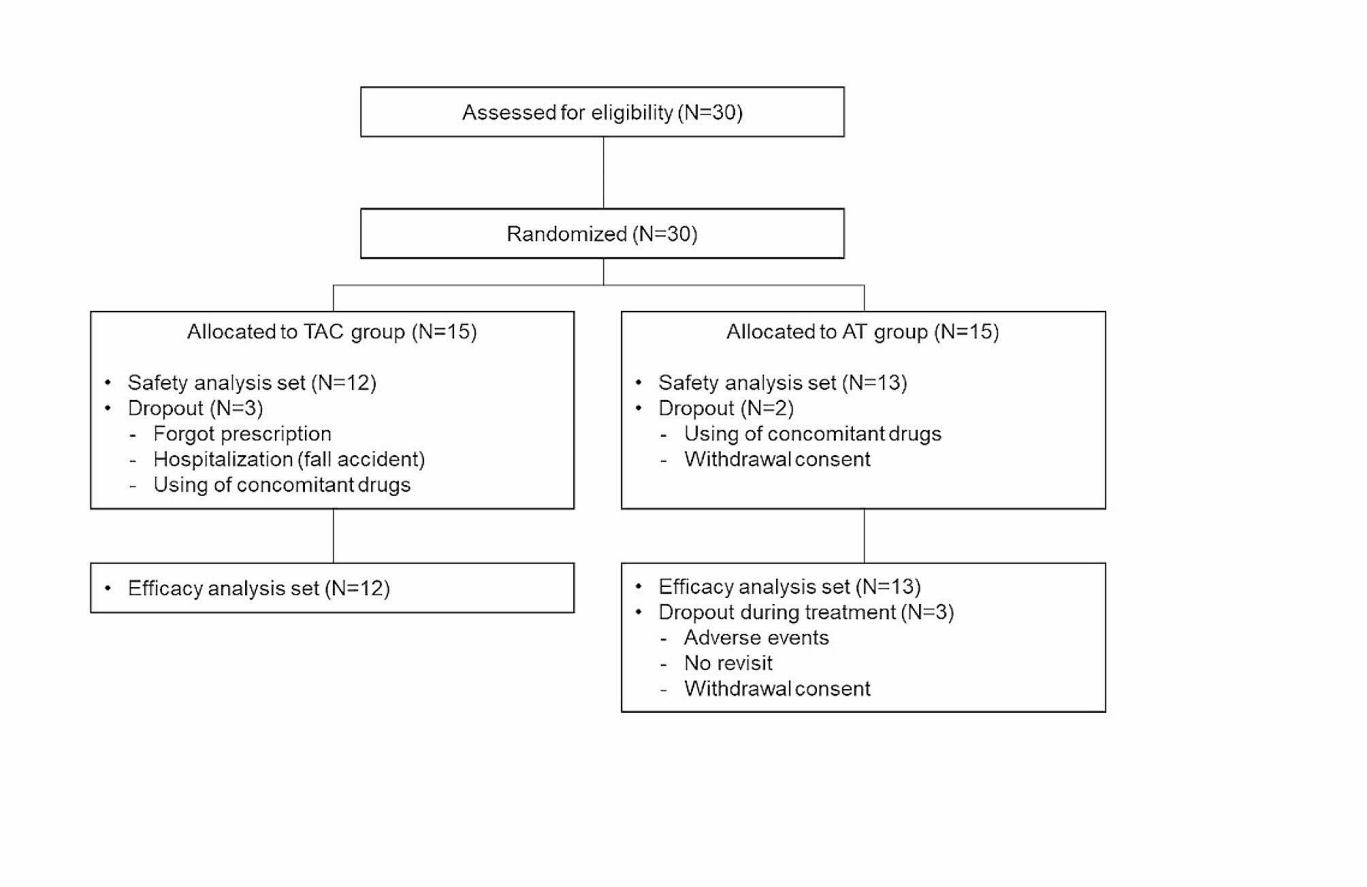
Study flowchart. TAC: tacrolimus, AT: artificial tears

**Fig. 2:**
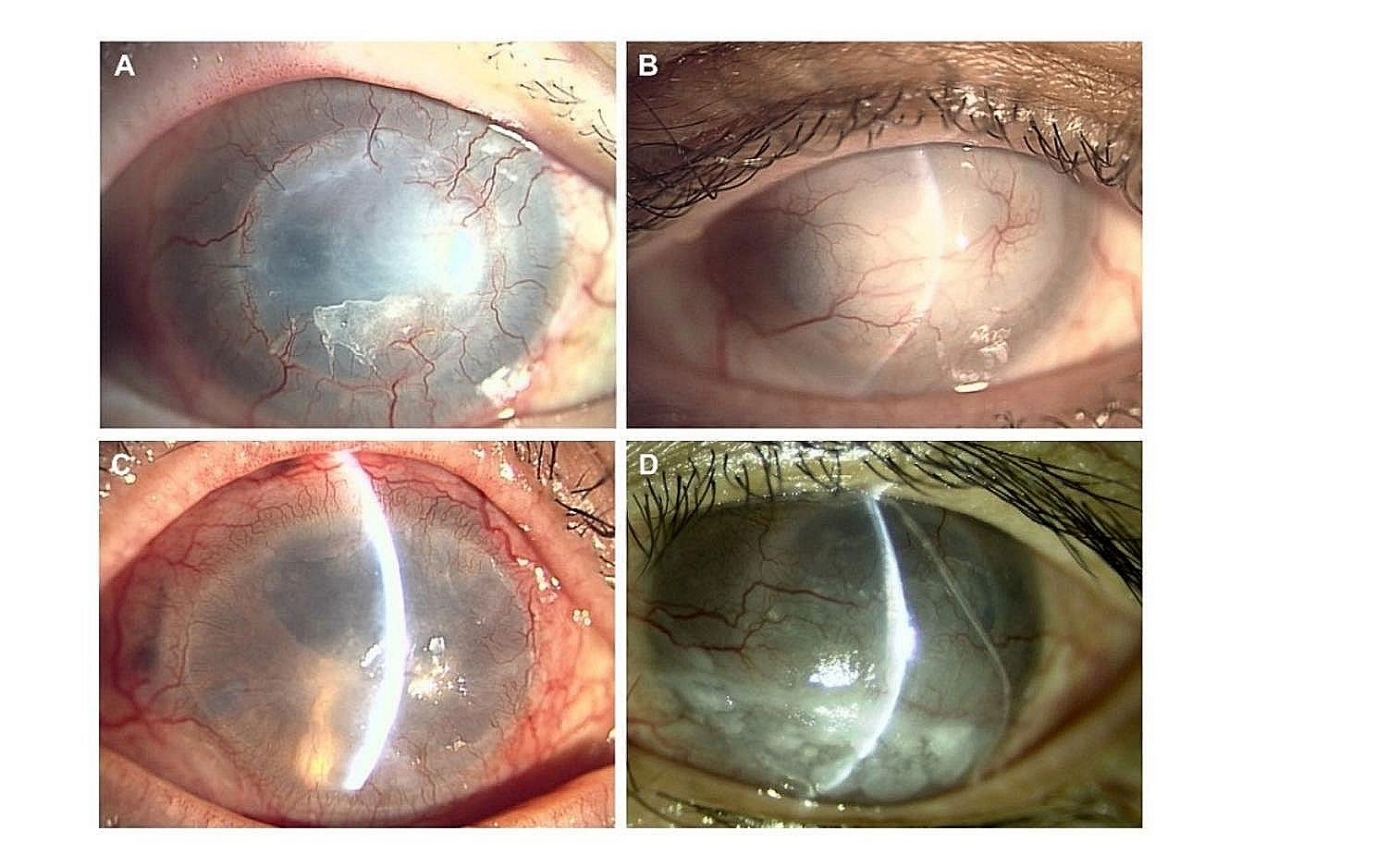
A preoperative anterior segment photograph of representative high-risk cases. Patients who had history of PKP (**A**-**B**), and patients who had history of PKP with deep corneal vascular invasion more than two quadrants (**C**-**D**). These patients had corneal opacity due to decompensation

Thirty patients were enrolled in this study, five of whom discontinued participation after providing informed consent. Therefore, 25 eyes were included in the study. The study flow diagram is shown in Fig. 1. There were 12 patients in the TAC group (11 men and one woman, median age: 67 years) and 13 patients in the artificial tear (AT) group (8 men and 5 women, median age: 67 years). All eyes in the TAC group and 11 eyes in the AT group had a history of previous corneal transplantation, and two eyes in the AT groups, had deep stromal neovascularization > 2 quadrants. A preoperative anterior segment photograph of a typical case in this study was shown (Fig. 2). There were no significant differences in any of the patient characteristics between the TAC and AT groups (Table 1). Summary of reasons for surgery in high-risk patients were shown in Table 2.

**Table 1:** Summary of participants’ baseline demographic and ocular characteristics

	Tacrolimus group (*N* = 12)	Artificial tear group (*N* = 13)
Age, years		
Mean (SD)	61.4 (17.7)	65.6 (11.6)
Median	67	67
Sex, N (%)		
Male	11 (91.7)	8 (61.5)
Female	1 (8.3)	5 (38.5)
Surgery eye, N (%)		
Right	5 (41.7)	6 (46.2)
Left	7 (58.3)	7 (53.9)
BSCVA, logMAR		
Mean (SD)	1.73 (0.72)	1.88 (0.82)
IOP, mmHg		
Mean (SD)	16.4 (4.6)	18.6 (7.2)

BSCVA: best spectacle-corrective visual acuity, logMAR: logarithm of the minimum angle of resolution, IOP: intraocular pressure, SD: standard deviation

**Table 2:** Summary of cause of corneal opacity in high-risk patients

	Cause of corneal opacity	Tacrolimus group (*N* = 12)	Artificial tear group (*N* = 13)
Regraft, N (%)		9 (75.0)	7 (53.8)
	Decompensation	6	3
	Decompensation following infectious keratitis	1	0
	Decompensation following rejection	1	0
	Decompensation following wound dehiscence	0	2
	Recurrence of lattice dystrophy	0	1
	Stromal scar	1	1
NV (> 1/2), N (%)		0	2 (15.4)
	Acanthamoeba infection	0	1
	Traumatic scar	0	1
Regraft + NV (> 1/2), N (%)		3 (25.0)	4 (30.8)
	Calcium deposits	1	0
	Decompensation	1	3
	Decompensation following infectious keratitis	1	0
	Stromal scar	0	1

NV (> 1/2): deep stromal neovascularization > 2 quadrants

### Immunological rejection

One patient in the AT group developed immunological rejection. This 53-year-old man underwent PKP plus extracellular cataract extraction due to a post-infectious keratitis scar associated with four-quadrant deep corneal neovascularization. He developed secondary glaucoma, which was controlled with medical treatment. The graft remained clear until week 13 after the surgery, when he developed endothelial rejection. Although the rejection was successfully treated with intensive topical steroid administration, the IOP was uncontrollable. The patient eventually lost his central visual field and the graft was decompensated. No episodes of rejection were observed in the TAC group. Survival curve analysis could not be performed because of the limited number of rejection episodes.

### Graft survival

**Fig. 3:**
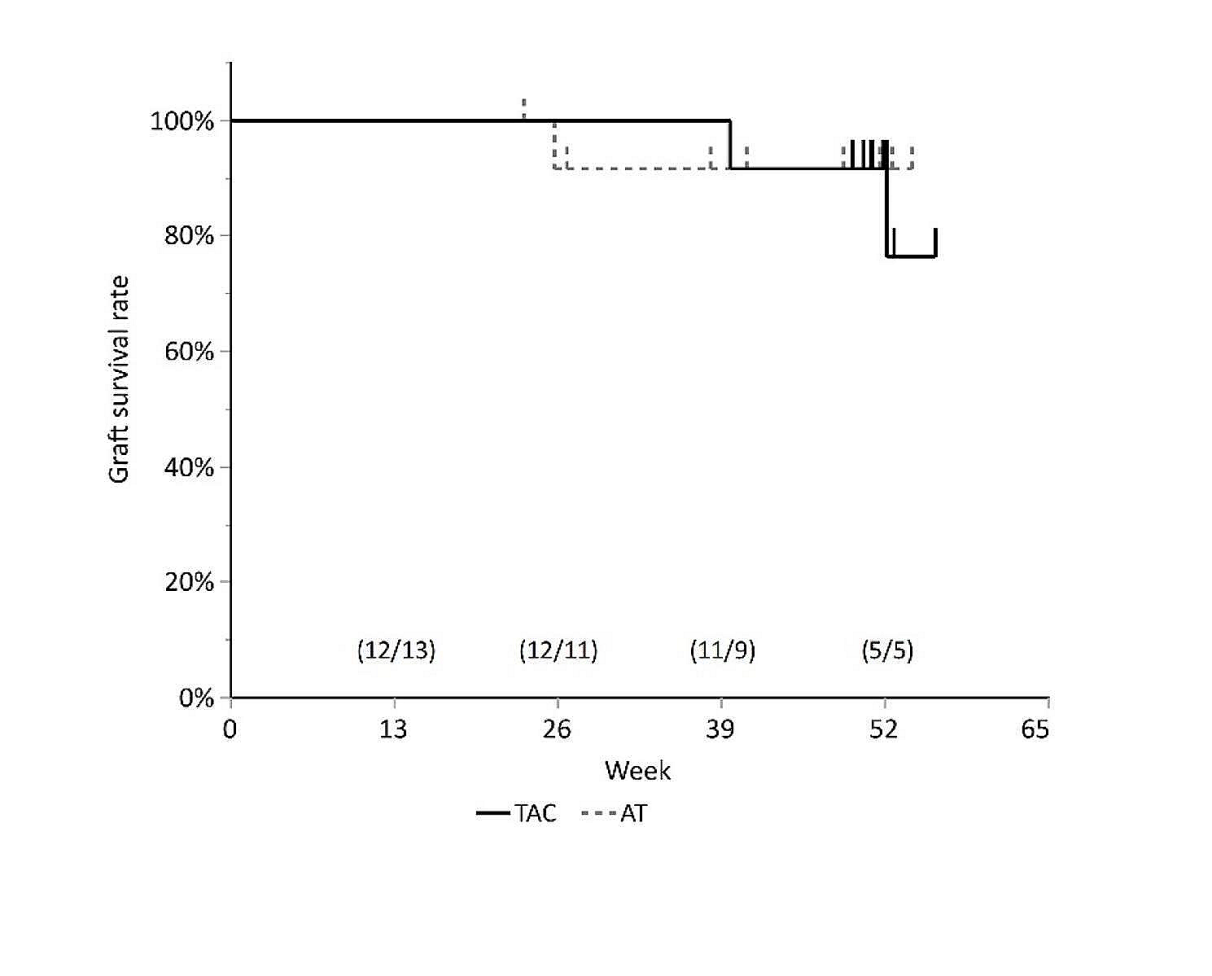
Kaplan-Meier curve of the graft survival rate. Log-rank test was employed to compare the incidence of rejection and graft survival rate between the groups (*P*=0.60). The number of patients at risk is shown as (TAC/AT) in the graph. TAC: tacrolimus, AT: artificial tears

Figure 3 shows the graft survival rates in the two groups. In the TAC group, the survival rates were 100% at weeks 13 and 26, and 91.7% and 73.3% at weeks 39 and 52, respectively. In the AT group, the graft survival rates were 100% at week 13, and 91.7% at weeks 26, 39, and 52. There were no significant differences in the graft survival rates between the groups (log-rank test, *P* = 0.60).

### Best spectacle-corrected visual acuity

**Fig. 4:**
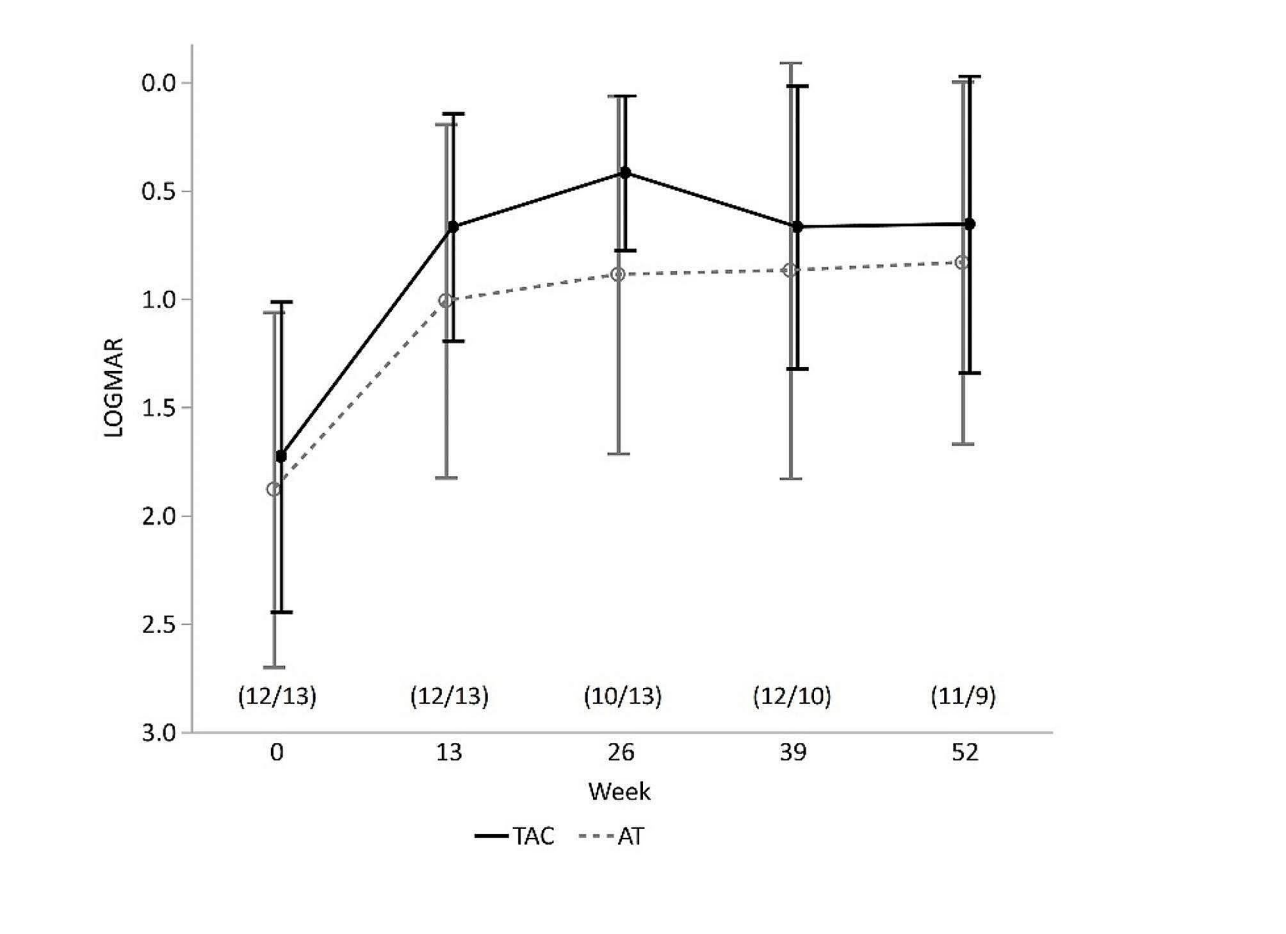
Mean change from baseline in BSCVA (logMAR)± SD. Student’s t-test was employed for statistical analysis. The number of patients used for analysis is shown as (TAC/AT) in the graph. TAC: tacrolimus, AT: artificial tears, BSCVA: best spectacle-corrected visual acuity, SD: standard deviation

Figure 4 shows the changes in the BSCVA in the two groups. At weeks 13, 26, 39, and 52 after surgery, the BSCVA was 0.67 ± 0.52, 0.42 ± 0.36, 0.67 ± 0.65, and 0.66 ± 0.69 in the TAC group and 1.01 ± 0.82, 0.89 ± 0.83, 0.87 ± 0.96, and 0.83 ± 0.84 in the AT group, respectively. There was no significant difference between the two groups throughout the observation period (Mann-Whitney two sample u test, P values at weeks 13, 26, 39, and 52 were 0.34, 0.11, 0.79, and 0.59, respectively).

### Corneal endothelial cell density

**Fig. 5:**
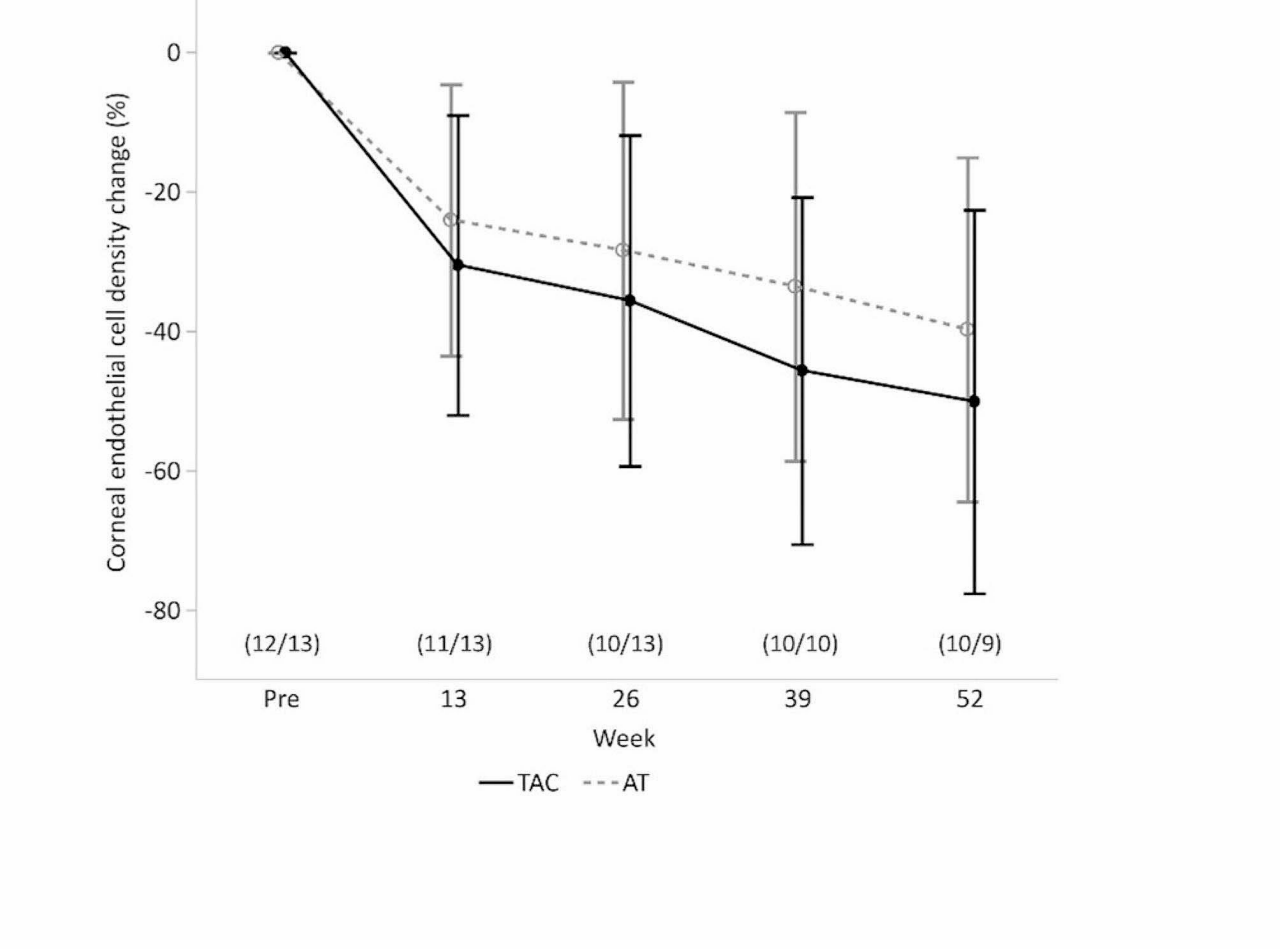
Mean change rate from baseline in corneal endothelial cell density (%) ± SD. Student’s t-test was employed for statistical analysis. The number of patients used for analysis is shown as (TAC/AT) in the graph. TAC: tacrolimus, AT: artificial tears, SD: standard deviation

Figure 5 shows the changes in the corneal ECD in the two groups. At weeks 13, 26, 39, and 52 after surgery, the rates of ECD loss were 30.5 ± 21.5%, 35.7 ± 23.8%, 45.7 ± 24.9%, and 50.2 ± 27.6% in the TAC group and 24.1 ± 19.5%, 28.4 ± 24.2%, 33.6 ± 25.1%, and 39.8 ± 24.7% in the AT group, respectively. There were no significant differences between the two groups throughout the observation period (Mann-Whitney two sample u test, P values at weeks 13, 26, 39, and 52 were 0.38, 0.40, 0.38, and 0.39, respectively)

### IOP changes

At week 52 after surgery, the number of cases with an IOP increase of ≥ 5 mmHg and ≤ 5 mmHg were one and 10 in the TAC group, and three and six in the AT group, respectively. There was no significant difference between the two groups (Fisher’s exact test, *P* = 0.28).

### Corneal epithelial damage

At week 52 after surgery, the number of patients with (score ≥ 1) or without corneal disorders (score 0) was four and seven in the TAC group, and none and nine in the AT group, respectively. No significant differences were noted between the groups in the occurrence of corneal disorders (Fisher’s exact test, *P* = 0.09).

### Adverse events

Table 3 summarises the adverse events. In the TAC group, adverse events were reported in three patients (25.0%), and included herpetic keratitis, corneal oedema, hyphema, corneal epithelial defect, and corneal plaque in one eye each. In the AT group, adverse events were reported in four patients (30.8%), and included fungal keratitis, macular oedema, ocular hypertension, graft rejection, and cataract development in one eye each. There was no significant difference between the two groups (Fisher’s exact test; *P* = 1.00).

**Table 3:** Summary of ocular adverse events

	Tacrolimus group (*N* = 12)	Artificial tear group (*N* = 13)
Total cases, N (%)	3 (25.0)	4 (30.8)
Herpetic keratitis, N (%)	1 (8.3)	0
Macular oedema, N (%)	0	1 (7.7)
Corneal plaque, N (%)	1 (8.3)	0
Graft rejection, N (%)	0	1 (7.7)
Corneal epithelial defect, N (%)	1 (8.3)	0
Ocular hypertension, N (%)	0	1 (7.7)
Corneal oedema, N (%)	1 (8.3)	0
Fungal keratitis, N (%)	0	1 (7.7)
Hyphema, N (%)	1 (8.3)	0
Cataract, N (%)	0	1 (7.7)

## Discussion

The present study was the first trial to investigate the efficacy and safety of the commercially available 0.1% topical TAC for the prevention of immunological rejection following high risk PKP. The study demonstrated that topical TAC was well tolerated in all patients, but failed to demonstrate positive effects in terms of prevention of immunological rejection. In other endpoints, such as graft survival, BSCVA, corneal ECD, IOP changes, and corneal epithelial damage, there was no significant difference between TAC and AT group. On the other hand, since no unknown or serious side effect was expressed in TAC group, tolerability for long-term use of TAC was demonstrated.

TAC has shown positive effects in facilitating long-term allograft survival compared to CsA in kidney and liver transplantation [22]. Several studies, including ours, have shown favourable preventive effects of systemic TAC on graft failure in high-risk corneal transplantation [17, 23, 24]. Despite its efficacy, systemic immunosuppression is associated with a relatively high incidence of side effects. For example, our previous study using systemic CsA in high-risk corneal transplantation revealed that systemic side effects developed in 25% of the patients [12]. Although TAC seems to have fewer side effects, this remains a significant problem [17, 25]. Many patients undergoing corneal transplantation are of older age, and accordingly, have a relatively high risk of side effects.

Several studies have shown that topical TAC is effective in preventing corneal graft rejection [18–20, 26–30]. Reinhard et al. reported that topical TAC was at least as effective as topical steroids in patients with normal-risk PKP [20]. Dhaliwal et al. reported that there was no irreversible graft rejection during topical TAC treatment for high-risk PKP [29]. Topical TAC also has safety advantages. Magalhaes et al. reported that topical TAC was effective in preventing irreversible rejection without increasing the IOP [18]. However, it should be noted that these studies used different concentrations of TAC with different study designs. This inconsistency makes the comparison difficult.

In the present study, all patients tolerated topical TAC administration. There were no significant differences in ocular AEs between the two groups. In a clinical trial of TAC for severe allergic conjunctivitis, 43% of patients reported ocular irritation [31]. The lower incidence of irritation symptoms in our study may be due to the older age of patients, resulting in a lower degree of ocular surface inflammation compared with that in the above study, which reported severe allergic inflammation in younger adults.

Despite the good tolerability, we did not observe positive effects of topical TAC in terms of prevention of immunological rejection following high-risk PKP. Only one eye in the AT group developed immunological rejection, and no such episodes were observed in the TAC group. There are several possible explanations for this finding. First, the sample size may not have been large enough to determine the difference in the effect of TAC. Although we recruited 30 patients (15 in each group), eight did not complete the study. This relatively large dropout rate may obscure the effect of TAC. Second, the observation period may not have been long enough to detect rejection episodes. It has been documented that more than half of rejection episodes develop within 1 year following corneal transplantation, and administration of immunosuppressive medications such as TAC may prolong rejection development. However, as we followed up the study patients, only one out of eight eyes in each group developed rejection after the observation period with the use of topical 0.1% fluorometholone, suggesting that it is unlikely that we would observe more rejection cases with a longer follow-up period. Third, the criteria for high-risk PKP may not have been sufficiently strict. Although many studies have indicated that previous grafting is a significant risk factor for immunological rejection [5, 32], a history of immunological rejection in previous grafting may be a stronger risk factor. In addition, some studies have indicated that the number of previous grafts is one of the risk factors for graft failure [33, 34]. All patients enrolled in the present study had neovascularization and/or a history of previous grafting; however, only three eyes had a history of rejection. Further clinical studies are needed to draw conclusions on the efficacy of topical TAC in high-risk PKP.

## Conclusion

Our results demonstrated that good tolerability of 0.1% TAC ophthalmic suspension in patients undergoing high-risk PKP. However, we failed to demonstrate its efficacy in preventing immunological rejection in our randomised clinical trial. Larger-scale clinical studies are required to draw conclusions on the efficacy of topical TAC in high-risk PKP.

## Data Availability

No datasets were generated or analysed during the current study.

## Abbreviations

PKP: Penetrating keratoplasty
TAC: Tacrolimus
AT: Artificial tear
CsA: Cyclosporine A
IOP: Intraocular pressure
BSCVA: Best spectacle-corrective visual acuity
ECD: Endothelial cell density
LogMAR: Logarithm of the minimum angle of resolution
